# Characteristics, treatments, and outcomes of severe sepsis of 3195 ICU-treated adult patients throughout Japan during 2011–2013

**DOI:** 10.1186/s40560-016-0169-9

**Published:** 2016-07-12

**Authors:** Mineji Hayakawa, Shinjiro Saito, Shigehiko Uchino, Kazuma Yamakawa, Daisuke Kudo, Yusuke Iizuka, Masamitsu Sanui, Kohei Takimoto, Toshihiko Mayumi, Takeo Azuhata, Fumihito Ito, Shodai Yoshihiro, Katsura Hayakawa, Tsuyoshi Nakashima, Takayuki Ogura, Eiichiro Noda, Yoshihiko Nakamura, Ryosuke Sekine, Yoshiaki Yoshikawa, Motohiro Sekino, Keiko Ueno, Yuko Okuda, Masayuki Watanabe, Akihito Tampo, Nobuyuki Saito, Yuya Kitai, Hiroki Takahashi, Iwao Kobayashi, Yutaka Kondo, Wataru Matsunaga, Sho Nachi, Toru Miike, Hiroshi Takahashi, Shuhei Takauji, Kensuke Umakoshi, Takafumi Todaka, Hiroshi Kodaira, Kohkichi Andoh, Takehiko Kasai, Yoshiaki Iwashita, Hideaki Arai, Masato Murata, Masahiro Yamane, Kazuhiro Shiga, Naoto Hori

**Affiliations:** Emergency and Critical Care Center, Hokkaido University Hospital, N14W5 Kita-ku, Sapporo, 060-8648 Japan; Intensive Care Unit, Department of Anesthesiology, Jikei University School of Medicine, Tokyo, Japan; Department of Emergency and Critical Care, Osaka General Medical Center, Osaka, Japan; Division of Emergency and Critical Care Medicine, Tohoku University Graduate School of Medicine, Sendai, Japan; Department of Anesthesiology and Critical Care Medicine, Jichi Medical University Saitama Medical Center, Saitama, Japan; Department of Critical Care, Shonan Kamakura General Hospital, Kamakura, Japan; Department of Anesthesiology and Intensive Care Medicine, Osaka University Graduate School of Medicine, Suita, Japan; Department of Emergency Medicine, University of Occupational and Environmental Health, Kitakyushu, Japan; Division of Emergency and Critical Care Medicine, Department of Acute Medicine, Nihon University School of Medicine, Tokyo, Japan; Department of Emergency and Critical Care Medicine, Ohta General Hospital Foundation, Ohta Nishinouchi Hospital, Koriyama, Japan; Pharmaceutical Department, JA Hiroshima General Hospital, Hiroshima, Japan; Department of Emergency and Critical Care Medicine, Saitama Red Cross Hospital, Saitama, Japan; Department of Emergency and Critical Care Medicine, Wakayama Medical University, Wakayama, Japan; Department of Emergency Medicine and Critical Care Medicine, Advanced Medical Emergency Department and Critical Care Center, Japan Red Cross Maebashi Hospital, Maebashi, Japan; Emergency and Critical Care Center, Kyushu University Hospital, Fukuoka, Japan; Department of Emergency and Critical Care Medicine, Faculty of Medicine, Fukuoka University, Fukuoka, Japan; Emergency Department, Ibaraki Prefectural Central Hospital, Kasama, Japan; Division of Intensive Care, Nagasaki University Hospital, Nagasaki, Japan; Department of Emergency and Critical Care Medicine, Tokyo Medical University, Hachioji Medical Center, Tokyo, Japan; Department of Emergency and Critical Care Medicine, Kyoto Daiichi Red Cross Hospital, Kyoto, Japan; Intensive Care Unit, Saiseikai Yokohamasi Tobu Hospital, Yokohama, Japan; Department of Emergency Medicine, Asahikawa Medical University, Asahikawa, Japan; Shock and Trauma Center, Nippon Medical School Chiba Hokusoh Hospital, Inzai, Japan; Emergency Medicine, Kameda Medical Center, Kamogawa, Japan; Department of Traumatology and Acute Critical Medicine, Osaka University Graduate School of Medicine, Suita, Japan; Emergency and Critical Care Medicine, Asahikawa Red Cross Hospital, Asahikawa, Japan; Department of Emergency and Critical Care Medicine, Graduate School of Medicine, University of the Ryukyus, Nishihara, Japan; Advanced Critical Care Center, Gifu University Hospital, Gifu, Japan; Emergency and Critical Care Center, Saga University Hospital, Saga, Japan; The Division of Cardiovascular Disease, Steel Memorial Muroran Hospital, Muroran, Japan; Department of Emergency Medicine and Critical Care, Sapporo City General Hospital, Sapporo, Japan; Division of Emergency Medicine, Ehime University Hospital, Toon, Japan; Intensive Care Unit, Tomishiro Central Hospital, Tomishiro, Japan; Department of Emergency Medicine, Akashi City Hospital, Akashi, Japan; Department of Emergency and Critical Care, Sendai City Hospital, Sendai, Japan; Emergency Department, Hakodate Municipal Hospital, Hakodate, Japan; Emergency and Critical Care Center, Mie University Hospital, Tsu, Japan; Department of Emergency Medicine, Gunma University, Maebashi, Japan; Department of Anesthesia and Intensive Care, KKR Sapporo Medical Center, Sapporo, Japan; Emergency and Critical Care Center, Seirei Mikatahara General Hospital, Hamamatsu, Japan; Intensive Care Unit, Hyogo College of Medicine, Nishinomiya, Japan

**Keywords:** Severe sepsis, Mortality, Epidemiology, Acute respiratory failure, Acute kidney injury, Disseminated intravascular coagulation, Organ failure, Septic shock

## Abstract

Severe sepsis is a major concern in the intensive care unit (ICU), although there is very little epidemiological information regarding severe sepsis in Japan. This study evaluated 3195 patients with severe sepsis in 42 ICUs throughout Japan. The patients with severe sepsis had a mean age of 70 ± 15 years and a mean Acute Physiology and Chronic Health Evaluation II score of 23 ± 9. The estimated survival rates at 28 and 90 days after ICU admission were 73.6 and 56.3 %, respectively.

**Electronic supplementary material:**

The online version of this article (doi:10.1186/s40560-016-0169-9) contains supplementary material, which is available to authorized users.

## Background

Many recent multicenter epidemiological studies have evaluated sepsis [1–7], although there is very little information regarding its epidemiology in Japan [1, 2]. Despite the limited amount of Japanese information, epidemiological data regarding severe sepsis are important for guiding clinical practice and the design of clinical studies. Therefore, the present study aimed to retrospectively evaluate a large population of patients with severe sepsis in intensive care units (ICUs) throughout Japan.

## Methods

The present study analyzed the unlinkable anonymized database of the Japan Septic Disseminated Intravascular Coagulation (JSEPTIC DIC) study [8]. Cases of shock, respiratory failure, or renal failure were defined as patients with a cardiovascular, respiratory, or renal Sequential Organ Failure Assessment (SOFA) score of ≥4 on day 1 [9]. Cases of disseminated intravascular coagulation (DIC) were defined as patients with a Japanese Association for Acute Medicine DIC score of ≥4 on day 1. All data were expressed as number (percent), mean ± standard deviation, or median (interquartile range), as appropriate. Survival rates were evaluated using the Kaplan-Meier method. All analyses were performed using SPSS software (version 22; SPSS Inc., Chicago, IL).

## Results

**Fig. 1:**
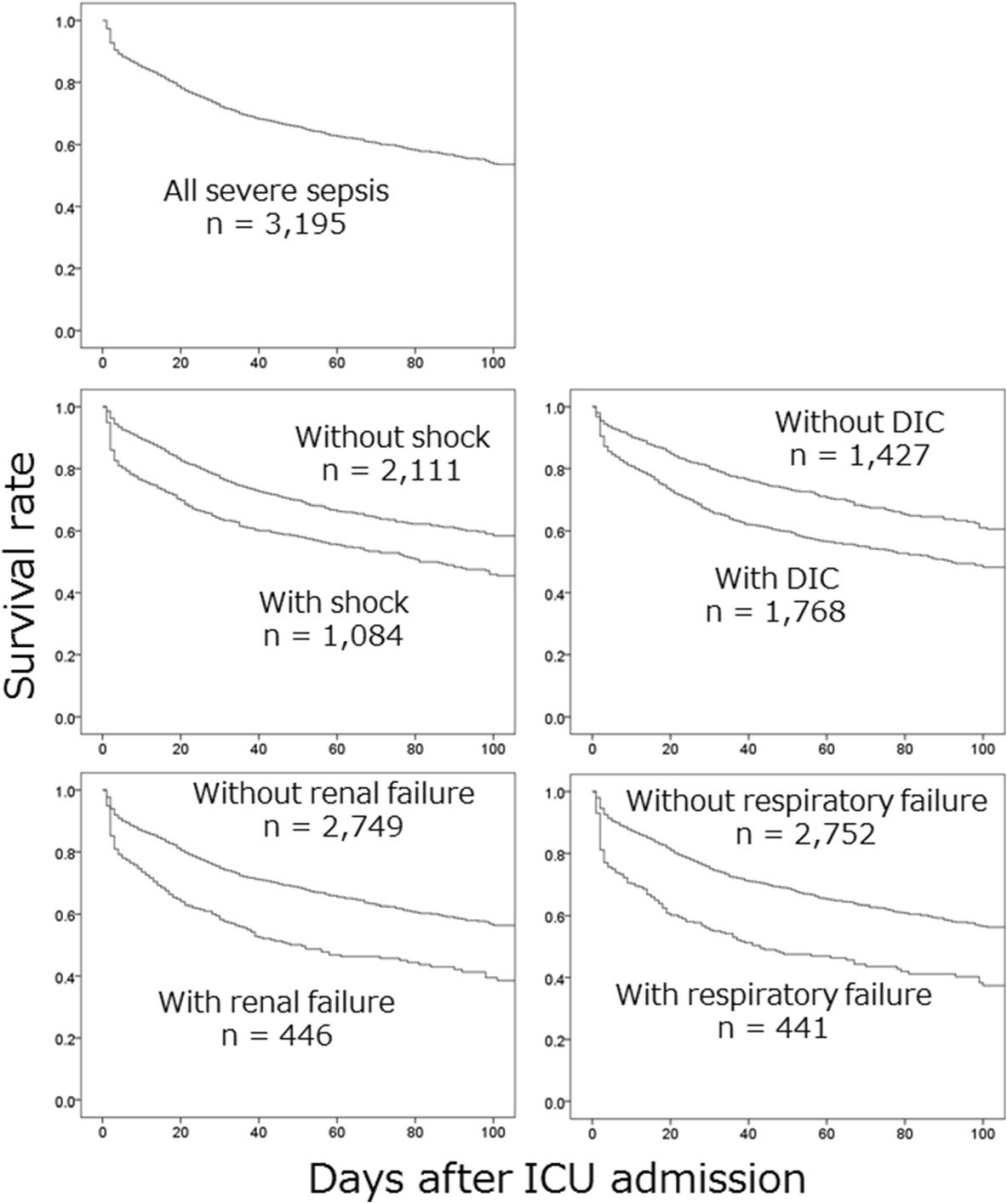
Survival curves for patients with and without various medical conditions. The patients with medical conditions exhibited a poorer survival rate, compared to the patients without the conditions. Cases of shock, respiratory failure, or renal failure were defined as a cardiovascular, respiratory, or renal Sequential Organ Failure Assessment (SOFA) score of ≥4 on day 1. Cases of disseminated intravascular coagulation (DIC) were defined as a DIC score of ≥4 on day 1

The present study included 3195 consecutive patients (2111 patients without shock and 1084 patients with shock). These patients included 1916 men (mean age 68 ± 14 years) and 1279 women (mean age 71 ± 15 years). The mean Acute Physiology and Chronic Health Evaluation II score among all patients was 23 ± 9. The primary infection sites are presented in Table 1. The blood culture results and responsible microorganisms are presented in Table 2. The frequencies of administering various adjunct treatments for severe sepsis during the first 7 days after ICU admission are shown in Table 3. The survival curves for patients with and without various medical conditions are presented in Fig. 1. The estimated survival rates at 28 and 90 days among all patients with severe sepsis after the ICU admission were 73.6 and 56.3 %, respectively.

**Table 1:** Primary infection site responsible for the sepsis

	Without shock	With shock	Total
	*n* = 2111	*n* = 1084	*n* = 3195
Abdomen	661 (31 %)	371 (34 %)	1032 (32 %)
Lung/thorax	575 (27 %)	252 (23 %)	827 (26 %)
Urinary tract	349 (17 %)	160 (15 %)	509 (16 %)
Bone/soft tissue	251 (12 %)	123 (11 %)	374 (12 %)
Cardiovascular system	54 (3 %)	14 (1 %)	68 (2 %)
Central nervous system	44 (2 %)	19 (2 %)	63 (2 %)
Catheter-related	23 (1 %)	21 (2 %)	44 (1 %)
Other	37 (2 %)	23 (2 %)	60 (2 %)
Unknown	117 (6 %)	101 (9 %)	218 (7 %)

Data are expressed as number (percent)

**Table 2:** Microorganisms responsible for the sepsis and blood culture results

	Without shock	With shock	Total
	*n* = 2111	*n* = 1084	*n* = 3195
Microorganisms responsible for the sepsis			
Gram-negative rod	774 (35%)	421 (39%)	1165 (37%)
Gram-positive coccus	477 (23%)	261 (24%)	738 (23%)
Fungus	43 (2%)	14 (1%)	57 (2%)
Virus	20 (1%)	8 (1%)	28 (1%)
Mixed infection	254 (12%)	146 (14%)	400 (13%)
Other	40 (2%)	18 (2%)	58 (2%)
Unknown	533 (25%)	216 (20%)	749 (23%)
Blood culture			
Positive	866 (41%)	540 (50%)	1406 (44%)
Negative	1,083 (51%)	508 (47%)	1591 (50%)
Not taken	162 (8%)	36 (3%)	198 (6%)

Data are expressed as number (percent)

**Table 3:** Frequencies of various adjunct treatments for severe sepsis during the first 7 days after the ICU admission

Adjunct treatments	
DIC treatments	1498 (47%)
Antithrombin	990 (31%)
Thrombomodulin	856 (27%)
Co-administration of antithrombin and thrombomodulin	496 (16%)
Protease inhibitors	392 (12%)
Heparinoids	167 (5%)
Immunoglobulin	976 (31%)
Low-dose steroids	777 (24%)
Renal replacement therapy	890 (28%)
Non-renal indication renal replacement therapy	266 (8%)
Polymyxin B-direct hemoperfusion	692 (22%)

Data are presented as number (percentage)

*DIC* disseminated intravascular coagulation, *ICU* intensive care unit

## Discussion

The present study evaluated the characteristics, treatments, and outcomes from 3195 patients with severe sepsis in 42 ICUs throughout Japan. The earlier epidemiological reports from after 2005 are summarized in the Additional file 1: Table S1. Although two previous Japanese studies have reported epidemiological information from 890 Japanese patients with severe sepsis, most of the participating institutions were university hospitals [1, 2]. In contrast, approximately half of the participating institutions in the present study were municipal hospitals. Furthermore, we included both general and emergency ICUs. Nevertheless, the distributions of age, severity, and mortality rates in the present study were similar to the findings from two previous Japanese studies [1, 2].

Patients with severe sepsis in other countries are generally younger than their Japanese counterparts [1–7]. Furthermore, other countries have higher mortality rates for patients with severe sepsis, compared to the rate from the present study, although the Acute Physiology and Chronic Health Evaluation II scores are similar for Japanese patients and other patients with sepsis [1–7]. However, the reports from the other countries evaluated patients with sepsis during an earlier period (2002–2010), compared to the patients from the three Japanese reports (2007–2013) [1–7]. Furthermore, mortality among patients with sepsis has decreased on an annual basis, and these factors may explain the different mortality rates in Japan and other countries.

The present study’s mortality rates for severe sepsis with and without shock are similar to the results from previous Japanese studies [1, 2]. However, severe sepsis is frequently complicated by respiratory failure, renal failure, and DIC [10], and the previous studies did not evaluate the mortality rates for severe sepsis in cases with respiratory or renal failure [1, 2]. Thus, the present study provides the first survival curve data for Japanese patients with severe sepsis according to their complications with shock, respiratory failure, renal failure, or DIC.

## Abbreviations

DIC, disseminated intravascular coagulation; ICU, intensive care unit; JSEPTIC DIC, Japan Septic Disseminated Intravascular Coagulation; SOFA, Sequential Organ Failure Assessment

## Additional file

Additional file 1: Table S1. Epidemiological information from previous reports after 2005. (DOC 48 kb)
