# Supplementary material for: Characteristics, treatments, and outcomes of severe sepsis of 3195 ICU-treated adult patients throughout Japan during 2011–2013
Source: J Intensive Care. 2016 Jul 12;4:44. doi: 10.1186/s40560-016-0169-9 (PMC4942911; doi:10.1186/s40560-016-0169-9)
Supplement: Additional file 1: Table S1. — Epidemiological information from previous reports after 2005. (DOC 48 kb) [file 40560_2016_169_MOESM1_ESM.doc]

|  | Duration | Number | Age | APACHE II score | SOFA score | In-hospital mortality rate |
| --- | --- | --- | --- | --- | --- | --- |
| From Japan |  |  |  |  |  |  |
| Present report | 2011–2013 | 3,195 | 70 ± 15 | 23 ± 9 | 9 ± 4 | 33% |
| JAAM [2] | 2010 | 624 | 69 ± 17 | 23 ± 8 | 9 ± 4 | 30% |
| JSICM [3] | 2007 | 266 | 67 ± 15 | 20 ± 10 | 9 ± 5 | 38% |
| From other countries |  |  |  |  |  |  |
| China [4] | 2009 | 484 | 66 (51–77) | 21 (16–27) | 8 (5–10) | 34% |
| Taiwan [5] | 2009–2010 | 536 | 64 ± 15 | NA | 8 ± 3 | 61% (at 28 days) |
| China [7] | 2004–2005 | 318 | 64 | 19 (14–25) | 8 (6–12) | 49% |
| Finland [8] | 2004–2005 | 470 | 60 | 24 ± 9 | NA | 28% |
| PROGRESS Registry [6] | 2002–2005 | 12,881 | 60 ± 18 | 23 ± 8 | 9 ± 4 | 50% |
| Germany |  | 1,855 | 64 ± 15 | 27 ± 8 | 11 ± 4 | 43% |
| Argentina |  | 1,269 | 61 ± 19 | 23 ± 8 | 7 ± 4 | 57% |
| Canada |  | 1,215 | 61 ± 16 | 23 ± 8 | NA | 50% |
| Brazil |  | 969 | 61 ± 18 | 23 ± 8 | 9 ± 4 | 67% |
| India |  | 803 | 55 ± 18 | 20 ± 7 | 10 ± 4 | 39% |
| USA |  | 761 | 62 ± 17 | 26 ± 8 | 11 ± 4 | 43% |
| Australia |  | 669 | 57 ± 18 | 21 ± 8 | 10 ± 4 | 33% |
| Malaysia |  | 641 | 50 ± 18 | 24 ± 9 | 10 ± 4 | 66% |

JSICM, Japanese Society of Intensive Care Medicine; JAAM, Japanese Association for Acute Medicine; PROGRESS, Promoting Global Research Excellence in Severe Sepsis; NA, not applicable.
Data are presented as mean ± standard deviation or median (interquartile range).
